# An update on captive cetacean welfare

**DOI:** 10.7717/peerj.19878

**Published:** 2025-10-31

**Authors:** Lori Marino, Catherine Doyle, Heather Rally, Lester O’Brien, Mackenzie Tennison, Bob Jacobs

**Affiliations:** 1 Whale Sanctuary Project, Washington, DC, United States of America; 2 Animal Studies Program, New York University, New York, NY, United States of America; 3 Performing Animal Welfare Society, San Andreas, CA, United States of America; 4 Foundation to Support Animal Protection, Norfolk, VA, United States of America; 5 Palladium Elephant Consulting Inc., Calgary, Alberta, Canada; 6 Department of Psychology, University of Washington, Seattle, WA, United States of America; 7 Neuroscience Program, Colorado College, Colorado Springs, CO, United States of America

**Keywords:** Captivity, Welfare, Cetacean, Dolphin, Whale, Marine park, Aquarium, Zoo

## Abstract

The welfare of captive cetaceans (*i.e.*, dolphins, whales, and porpoises) has garnered increasing attention over the years as captivity presents significant challenges for these long-lived, highly intelligent, wide-ranging, and socially complex animals. The present paper provides an overview of the current state of captive cetacean welfare, examining captive facilities, recent improvements, persistent problems, and the clinical/behavioral/neural consequences of confinement. We specifically address both quantitative and qualitative aspects of captive space, sociocognitive factors, feeding, and welfare concerns such as stereotypies, physical health, reproduction, and lifespan. The contrast between the restrictive nature of captive environments and the dynamic, multifaceted characteristics of the natural environment highlights the difficulties faced by cetaceans in captivity. Despite efforts by some facilities to improve conditions, serious welfare challenges persist, raising critical ethical concerns about the well-being of captive cetaceans.

## Introduction

Cetaceans (*i.e.,* dolphins, whales, and porpoises) have long been considered challenging and ethically problematic candidates for captivity (Carter, 1982; Goldblatt, 1993; Lott & Williamson, 2017; Rose et al., 2017; Hosey, Melfi & Ward, 2020; Marino, 2020; Marino et al., 2020; Marino & White, 2022; McGillian, 2023). Typically, only odontocetes (*i.e.,* toothed whales) are held in captivity because mysticetes (*i.e.,* baleen whales) are too large and have feeding habits that make captivity particularly challenging. The 2013 film *Blackfish,* directed by Gabriela Cowperthwaite, played a key role in changing public sentiment about captive cetaceans, particularly orcas (“killer whales”) by focusing on the disturbing personal history of the orca Tilikum (Parsons & Rose, 2018; Boissat, Thomas-Walters & Veríssimo, 2021). Increasingly, the public in many parts of the world has become more concerned about the welfare of captive cetaceans and less favorable towards captivity itself (Giovos et al., 2019; Naylor & Parsons, 2018). Wassermann, Hind-Ozan & Seaman (2018) found that, when accounting for some biases in previous surveys of visitor attitudes, support for orca shows and swim-with-dolphins (SWD) programs in their survey was well below what had been reported in previous studies conducted on behalf of captive cetacean attraction operators. Clegg (2021) argued that captive cetacean facilities will need to respond to these changing social ethics to survive into the future.

This increase in public concern has led to several legislative efforts around the world to limit or eliminate keeping cetaceans for entertainment. The U.S.-based SWIMS Act (2024), a federal bill to phase out captive cetacean entertainment in the United States, was last reintroduced in 2024. Canadian Bill S-203 (Parliament of Canada, 2025), passed in 2019, bans the breeding and use of captive cetaceans for pure entertainment. In 2021, the French government decreed a trade and breeding ban, which will end whale and dolphin captivity by 2026 (Berry, 2021). In 2024, Belgium officially became the seventh country worldwide and the fourth in Europe to enact a permanent ban on dolphinaria (The Brussels Times, 2024). Most recently, Mexico’s Senate approved a nationwide ban on the use of marine mammals in entertainment (Mexico Daily News, 2025). The above mentioned legislation requires alternative housing options be in place that would improve the animals’ welfare (see ‘Discussion and Conclusion’).

Nevertheless, captive cetacean entertainment persists across 50 countries in the world, with most facilities found in China, the U.S., Mexico (but see above), Japan, and Russia (Ceta-Base, 2025). The scope of the issue of captive cetacean welfare is broad. Globally, more than 3,500 cetaceans are housed in concrete tanks or sea pens/lagoons in ∼350 marine parks, zoos, and military facilities (Ceta-Base, 2025). The most abundant cetacean species in captivity are common bottlenose dolphins (*Tursiops truncatus,* ∼87% of all cetaceans; Brando et al., 2018), beluga whales (*Delphinapterus leucas*), and orcas (*Orcinus orca*), but also includes pilot whales (*Globicephala macrorhynchus*), Commerson’s dolphins (*Cephalorhynchus commersonii*), white-sided dolphins (*Lagenorhynchus obliquidens*), and several other species and hybrids (Ceta-Base, 2025). In North America alone, there are ∼480 bottlenose dolphins, ∼60 belugas, and 18 orcas in captivity (Ceta-Base, 2025).

Animals in zoos and marine parks in the US are covered by regulations set forth under the Animal Welfare Act (2023), which are often under-enforced (Winders & Chilakamarri, 2018). Accrediting organizations (*e.g.*, Association of Zoos and Aquariums, AZA; ∼40 members/facilities housing cetaceans) set welfare standards for their members. More broadly, some professional associations specifically focus on aquatic animals (*e.g.*, Alliance of the Marine Mammal Parks and Aquariums, AMMPA; ∼50 members; European Association for Aquatic Mammals, EAAM). In addition, the World Association of Zoos and Aquariums (WAZA; ∼400 members) provides a platform for collaboration among institutions and sets ethical guidelines but does not serve as an accrediting body. However, even under the highest standards, there remain unique challenges in meeting the essential needs of the animals (Morgan & Tromborg, 2007; Shepherdson & Carlstead, 2020). Moreover, only a relatively small number of facilities (∼2.3%; Catibog-Sinha, 2008; Bacon, 2018) appear to be accredited. For example, China has more than 100 facilities (30 added in just the last five years) holding dolphins (excluding orcas)—the largest number of any country (Ceta-Base, 2025). Only one facility there (*i.e.,* Ocean Park Corporation in Hong Kong) is accredited by the Association of Zoos & Aquariums (AZA) (2025). This indicates the potential for widespread problems in welfare for the majority of captive entertainment facilities.

Recently, a series of nine articles known as the Cetacean Welfare Study examined captive dolphin welfare measurements across 43 accredited zoos and aquariums in seven countries (Lauderdale et al., 2021a; Lauderdale et al., 2021b; Lauderdale et al., 2021c; Lauderdale et al., 2021d; Lauderdale et al., 2021e; Miller et al., 2021a; Miller et al., 2021b; Miller et al., 2021c; Miller et al., 2021d). It was a correlational study designed to identify factors related to the welfare of bottlenose dolphins (*Tursiops truncatus*), Indo-Pacific bottlenose dolphins (*Tursiops aduncus*), beluga whales (*Delphinapterus leucas*), and Pacific white-sided dolphins (*Lagenorhynchus obliquidens*). This project did not examine unaccredited facilities or those housing other cetacean species. These studies attempted to determine which welfare factors were most important for dolphins, and which ones could be addressed in the future. Identified factors included habitat characteristics and management practices (Lauderdale et al., 2021c; Lauderdale et al., 2021d; Miller et al., 2021b), environmental enrichment (Lauderdale et al., 2021e), health reference intervals (Lauderdale et al., 2021a), biomedical markers (Lauderdale et al., 2021a; Miller et al., 2021d) social behavior (Miller et al., 2021b), and behavioral diversity (Miller et al., 2021a). The findings of these studies are integrated in the present review where relevant.

The science of captive wild animal well-being continues to be informative about the needs of various species held in zoos, marine parks, and aquaria (Whitham & Wielebnowski, 2013; Clegg & Delfour, 2018; Shepherdson & Carlstead, 2020). Yet, several species, including cetaceans, still face significant challenges in captive settings (Clubb & Mason, 2003; Morgan & Tromborg, 2007; Mason, 2010; Fischer & Romero, 2018; Limin et al., 2025). Welfare assessment tools are used to measure how an animal is coping in its environment (Jones et al., 2022). There are numerous markers of poor welfare (*e.g.*, stereotypies, defined as unchanging repetitive actions; Mason, 1991; Jacobs et al., 2021) and, too, ways to determine positive welfare (Lauderdale et al., 2021b). The Cetacean Welfare Study, for example, found that environmental enrichment programs and social management factors were indicative of positive welfare in dolphins in accredited facilities (Lauderdale et al., 2021b). Importantly, good welfare is not simply equivalent to the absence of negative welfare markers (Miller et al., 2020). There are several factors such as diet, shelter, health, ability to express species-specific behaviors, and choice, that contribute to positive well-being (Vicino & Miller, 2015).

The objective of the present review is to examine the current well-being of captive cetaceans. To that end, we review the literature to assess how well the care of captive cetaceans around the world aligns with their needs, aiming to identify remaining challenges. This includes an overview of behavioral and clinical factors in cetacean captivity, particularly in managed settings. Our focus is on the most recent research and representative facilities, many (but not all) of which hold accreditation from one or more professional organizations, presumably indicating higher welfare standards, but we did not exclude unaccredited facilities. Although bottlenose dolphins in accredited facilities are the subject of most welfare assessments and most published literature, it is important to consider other species (*e.g.*, beluga whales, orcas) and to acknowledge that there are also many unaccredited facilities housing cetaceans around the world. Finally, we discuss the ethical implications of our findings.

## Literature Review Methodology

Our methodology involved conducting a scoping review of the literature (Munn et al., 2018) on captive cetacean welfare and related topics such as: body condition, dietary preferences, health, stereotypies, disease, maturation, sociality, reproduction, housing, management, enrichment, performance, space, complexity, and behavioral diversity. After a review of that literature, we examined aspects of the captive setting important to cetacean welfare: (a) enclosures, space and exercise, (b) complexity and sensory-perceptual experience, (c) sociality, (d) feeding and cognitive demands, and (e) performances and interactions with humans, as well as specific welfare topics: brain and behavior (*e.g.*, stereotypies, aggression) and physical health (*e.g.*, nutrition and metabolism, skin health, dental disease, digestive and gastrointestinal disease, infections, reproduction, longevity, survival rates, and mortality rates). We examined and included data on free-living and captive cetaceans.

We conducted our primary searches using Google Scholar. We also used our own papers and references from published scientific papers. Given that the field of cetacean welfare advances rapidly, we focused on articles published since 2000 with only a few exceptions. We used 314 substantiated sources of information in this review. These included 246 peer-reviewed papers, 25 chapters in edited books, and seven scientific books. We used 36 sources of gray literature (*e.g*., conference proceedings, white papers, government documents, accrediting organization websites). We gave priority to the most recently dated findings. Twenty citations were for publications prior to the year 2000, 199 published between 2000–2019, and 95 between 2020–2025. We obtained information about standards and recommended practices published by professional organizations from their websites. Moreover, we did not omit findings that demonstrated improvements in welfare or positive views of captive cetacean welfare because our aim was to generate an accurate, comprehensive and current picture of the status of captive cetaceans. To confirm that our search was comprehensive and inclusive, we examined reference lists of all articles we used. Finally, findings from secondary sources (*e.g.*, review articles, online web pages) were confirmed before being incorporated into the present study.

## The Captive Environment

Captivity in the present context is the state of being confined to an artificial environment (usually designed for human benefit/convenience), which is typified by zoos, aquaria, and marine parks but also includes research laboratories and military facilities (Hancocks, 2002; Morgan & Tromborg, 2007; Marino, 2018; Marino & White, 2022). Authentic sanctuaries (discussed in ‘Discussion and Conclusion’ section) are also a form of captivity designed for animals who cannot be released or returned to the wild (Doyle, 2017). They differ in that an authentic sanctuary prioritizes the well-being of each resident over human interests in an entirely non-exploitive setting more consistent with their evolutionary history and needs (Marino et al., 2025). Across these managed establishments, there are significant differences in terms of space allotted, quality of veterinary care, level of visitor interactions, inclusion of demonstrations/performances, and several other factors (Shepherdson & Carlstead, 2020). The captive environment is multifaceted, influencing both physical and behavioral aspects of welfare. Key factors include the size/complexity of enclosures, movement, sensory experiences and mental stimulation, diet, and social interactions.

### Enclosures, space, exercise

Approximately 81% of managed cetacean habitats are concrete pools, with the rest being either net sea pens or lagoons (Bruck, 2024; Ceta-Base, 2025). Individuals housed in enclosures continuous with the ocean tend to fare somewhat better than those in tanks (see below; Ugaz, Sánchez & Galindo, 2009; Ugaz et al., 2013). In the Cetacean Welfare Study, ∼58% of dolphins lived in zoo/aquarium habitats and ∼42% lived in habitats connected to ocean water (Lauderdale et al., 2021e). Cetaceans are also often managed by holding them in various pools for training, medical procedures, and to separate aggressive individuals. There are also quarantine pools and maternity pools that allow for separation and enhanced control over the animals. Moreover, some tanks have lifting floors that allow the animal to be lifted out of the water to be physically restrained by trainers and veterinarians (Couquiaud, 2005). The Cetacean Welfare Study found that most dolphin facilities had five or fewer areas that were separated by gates (Lauderdale et al., 2021e).

In this context, it is important to acknowledge that cetaceans have evolved for efficient, long-distance swimming (Buchholtz, 2001; Gillet, Frédérich & Parmentier, 2019), with certain species specialized for deep diving (Piscitelli et al., 2013; Hindle, 2020), activities that are severely curtailed in captivity. There is considerable variation in tank size across captive facilities. Many tanks are too small or shallow to allow natural swimming behaviors, especially for larger cetaceans (Lott & Williamson, 2017; Rose et al., 2017). For instance, orcas are the largest odontocetes held in captivity, with adult males ranging up to nine m in length and weighing close to 7,000 kg (Baird, 2002). The smallest orca tank at present appears to be at Mundo Marino, an aquarium in Argentina, and houses Kshamenk (∼six m long; 3,500 kg); the tank is ∼12 m long and ∼6.1–9.1 m deep. Orca tanks at SeaWorld facilities average ∼26.2 m by ∼15.5 m, with a depth of 10.4 m. One of the largest pools in the world housing orcas is the Port of Nagoya Public Aquarium in Japan. It is 60 m long and 30 m wide and 12 m deep, resulting in an overall water volume of ∼13,499 m^3^. By comparison, the Salish Sea, the primary home range to southern resident orcas, is ∼18,000 km^2^ in area and contains ∼2 × 10^12^ m^3^ of water (MacCready et al., 2021). Within their large home ranges, orcas “routinely swim multiple kilometers in straight lines and are capable of travelling as many as 225 km a day for up to 30–40 days without rest” (p. 46, Rose et al., 2017), reaching up to 9,400 km in 42 consecutive days (Durban & Pitman, 2012). To put this in perspective, a distance of 225 km would require ∼1,518 circular laps around the pool of the Nagoya Public Aquarium (or 5,357 circular laps at Mundo Marino aquarium). In terms of depth, the deepest recorded dive for an orca is 1,087 m (Towers et al., 2019), although they typically dive to much shallower depths (∼200–400 m; Miller, Shapiro & Deecke, 2010; Wright et al., 2017; Tennessen et al., 2019).

Similar ranges and depths have been documented for beluga whales as well. For example, the summer core range for the Eastern Beaufort Sea population has been estimated to be 36,349 km^2^; the actual home range is much larger, consisting of the Amundsen Gulf, the eastern Beaufort Sea shelf, shelf and slope regions west and north of Banks Island into M’Clure Strait and Viscount Melville Sound (Hauser et al., 2014). These whales are also known to swim >50 km/day (Hauser et al., 2014) and dive as deep as ∼900 m (Hauser et al., 2015).

For captive dolphins in the Cetacean Welfare Study, Lauderdale et al. (2021e) found that mean enclosure length was 41.28 m (width was not reported) and the mean maximum habitat depth was 7.66 m, allowing access to 2,610 m^3^ at night, and 2,540 m^3^ during the day. Bottlenose dolphin home ranges vary considerably, from 20 km^2^ to 344 km^2^ (Nekolny et al., 2017), and they are known to travel 33–89 km a day while making journeys of up to 4,200 km (Wells et al., 1999; Wells & Scott, 2009). Most captive bottlenose dolphins are of a coastal ecotype (*e.g.*, from habitats like Sarasota Bay) and typically stay in shallow waters (<four m; Wells et al., 2013). By comparison, offshore bottlenose dolphins regularly dive deeper than 500 m and sometimes as deep as 1,000 m (Fahlman et al., 2022). There does not appear to be any research on the daily distances covered by dolphins in captivity, although Lauderdale et al. (2021d) measured average distance traveled per hour (ADT). They found that captive dolphins traveled an average of 2.32 km/hr during the day, slightly above the rates of wild bottlenose dolphins on the Pacific Coast of the United States (Irvine et al., 1981). However, Lauderdale et al. (2021d) did not track movement at night, noting that captive dolphins, unlike their wild counterparts (Shorter et al., 2017), exhibit reduced activity at night. Using a 3D video-tracking system, Rachinas-Lopes et al. (2018) found that captive bottlenose dolphins spent most of their time (85%) at the surface in the deep area of their concrete pool, presumably because they have more stimuli (*e.g.*, trainers, objects, food) at the surface. They also suggested that dolphins in their natural habitat might have more incentive to explore deeper waters (Rachinas-Lopes et al., 2018). This would seem to be confirmed by the Cetacean Welfare Study, which found that dolphins in accredited ocean habitats swam in the top third of the water column less often than dolphins in managed zoo/aquarium tanks (which were deeper), perhaps because they were exploring the more natural ocean environment (Lauderdale et al., 2021b; Miller et al., 2021b).

Amount of space is a complex issue. Some early studies indicate that, when dolphins were held in unusually small pools, they were more likely to exhibit stereotypies (Greenwood, 1977) or exhibit a larger number of sexual/aggressive behaviors (Myers & Overstrom, 1978), but these behaviors disappeared when the dolphins were moved to a larger enclosure (Jaakkola, 2023). In more modern facilities, Lauderdale et al. (2021c) and Lauderdale et al. (2021d) found that environmental enrichment and predictable training schedules were more strongly associated with how dolphins used their habitat (*e.g.*, time spent in bottom third of enclosure) and with distance traveled (as measured by ADT) than were habitat characteristics (*e.g.*, tank size, water volume availability). Similarly, Miller et al. (2021b) and Miller et al. (2021c) also found that type and timing of enrichment can be more important than tank size for captive bottlenose dolphins. Specifically, captive dolphins on a more predictable schedule of enrichment activities were more socially interactive than those on a random schedule. These findings from the Cetacean Welfare Study would suggest that enclosure size is not a primary driver of the tested welfare outcomes for dolphins in the accredited facilities examined. However, given the orders of magnitude difference between captive pools and the extensive natural home ranges for which many cetaceans have evolved, there could be a potential confound (*i.e.,* a floor effect) with conclusions about how tank size in modern facilities affects movement and social behavior. In the Cetacean Research Study, for example, the magnitude of habitat change in length from the smallest measure to the largest is 13.1; in depth, it is 6.4 (Lauderdale et al., 2021e, Table 5). These are miniscule changes compared to the change from the captive to the natural habitat. Not only the size of a species’ native habitat for which it evolved, but also the actual size of a species (*e.g.*, a goldfish *vs.* an orca) may affect its perception of enclosure size/complexity (De Azevedo et al., 2023). As such, it could be that the different sizes and complexities of captive enclosures do not constitute a meaningful difference for cetaceans. Studies incorporating significantly larger areas could potentially find associations between enclosure size/complexity and welfare outcomes. In conclusion, the strong mismatch between their natural aquatic behavior and captive enclosures, along with the complexities of habitat issues themselves, indicate that welfare in captive cetaceans is impacted by an array of interwoven factors.

### Complexity and sensory-perceptual experience in tanks

In terms of the physical environment, sensory-perceptual experiences in a concrete tank are largely determined by the physical features of the enclosure, which are relatively limited and unchanging. Most captive cetacean tanks are painted light or bright blue and designed to maximize the ability of visitors to observe them. Underwater windows may also be present so that visitors can view the animals. To ensure ease of maintenance and cleaning, tanks have smooth concrete surfaces and substrates and are relatively featureless as opposed to naturalistic textures (Couquiaud, 2005). Water clarity and cleanliness is typically achieved through filtration, ozonation, and chlorination (Couquiaud, 2005). Remarking on the lack of complexity in captive environments and the potential for negative consequences, Jaakkola (2024, p.2) stated:

In other words, in contrast to the situation in the wild, these animals live in highly predictable and structured environments where their primary physical needs—food, shelter, and safety—are generally met. On the one hand, this can clearly be viewed as positive, as these animals’ life dependent challenges have been solved. Indeed, providing animals with adequate food, shelter, and safety is universally considered fundamental to ensuring their positive welfare...That said, however, there is also a potential question about how this might impact their psychological well-being. That is, if an animal’s mind evolved to solve problems, and those problems disappear, what is that mind supposed to do? And how does this impact the animal’s welfare?

The acoustic properties of concrete tanks, which are affected by its size, depth, surfaces, and configuration, can be problematic for cetaceans, who are highly reliant on sound to perceive/navigate their environment (Au, 1993; Pack et al., 2002; Branstetter & Mercado, 2006), and for socialization/communication (Janik, Sayigh & Wells, 2006; Janik, 2014). Several alterations in cetacean vocal patterns have been noted in captivity in part because smooth tank surfaces, as opposed to rough or complex surfaces, result in a greater intensity of reverberations due to less scattering of the signal (Au, 1993). Captive humpback dolphins have been found to produce attenuated echolocation signals because of the reverberant environment of the tank (Niu et al., 2021). Irrawaddy dolphins exhibited a reduction in whistles after years in captivity (Svarachorn et al., 2016). Following transportation to a new facility, beluga whales exhibited a significant and prolonged reduction in vocalizations, suggesting stress-related suppression in acoustic activity (Castellote & Fossa, 2006). The effects of a captive environment on cetacean acoustic behavior is an area where additional research is needed (Stevens, Hill & Bruck, 2021), with some researchers suggesting that acoustic behavior could be used to evaluate cetacean welfare (Jones et al., 2021; Winship & Jones, 2023).

Problematic anthropogenic noise is found in both natural and captive environments (Duarte et al., 2021). In captivity, anthropogenic noise originates from a variety of sources (*e.g.*, nearby traffic, construction, amusement park rides, outdoor speakers, enrichment devices; Huettner et al., 2021; Stevens, Hill & Bruck, 2021). It has been shown to decrease dolphin play behavior, increase fast swimming behavior, and to decrease training performance (Huettner et al., 2021). Anthropogenic noise in captive settings has also been shown to alter acoustic behavior in bottlenose dolphins (Therrien et al., 2012) and to disrupt communication involved in performing a cooperative task (Sørensen et al., 2023). Persistent, anthropogenic noise, if not dampened sufficiently, can increase stress (Wright et al., 2007) and negatively impact welfare (*i.e.,* increase levels of cortisol, a stress hormone; Monreal-Pawlowsky et al., 2017; Yang et al., 2021) but more research is needed to determine how prevalent this issue is for captive cetaceans.

### Sociality

Although there is variability across cetacean species in terms of level and type of sociality (*i.e.,* the ways in which groups are structured and held together), the species usually kept in captivity (*e.g.*, common bottlenose dolphins, orcas, beluga whales) are highly social. They have extensive juvenile periods during which they learn cultural practices within a complex social network that is maintained throughout their lives in the wild (Williams & Lusseau, 2006; O’Corry-Crowe et al., 2020; Whitehead & Rendell, 2021). Bottlenose dolphins can form nested alliances within these social networks (King et al., 2018). Free-living orcas live in nested pods within clans that are bonded by dialect and other behavioral traditions (Williams & Lusseau, 2006; He, 2023). In the wild, beluga whales live in small groups that join, from time to time, with larger aggregations of hundreds or thousands of individuals (O’Corry-Crowe et al., 2020). Adult females are strongly bonded with their newborns as well as with older offspring. These triads stay together and join with others to form large nursery groups. Generally, group composition is fluid and underwritten by complex vocalizations and a variety of other characteristics (O’Corry-Crowe, 2009).

In captivity, by contrast, cetaceans live in managed collections that largely forgo opportunities for choice in relationships (Waples & Gales, 2002). Clegg & Butterworth (2017) noted that social group composition in captive facilities is somewhat artificial and under the control of zoo management rather than the animals themselves. Animal welfare scientists have stated that “keeping animals in appropriate social groupings, and with the required space and complexity to allow individuals to choose to spend time together or apart, is likely to be the most important welfare consideration” (Brando & Buchanan-Smith, 2018, p 85). After the minimum weaning age, captive cetacean mothers and offspring living in marine parks may be separated, as are other socially bonded individuals (Jett & Ventre, 2015). Because captive cetacean groups do not resemble social groups in the wild, there may be long-standing repercussions for the psychosocial and neural well-being of calves, as has been demonstrated in elephants (Bradshaw & Schore, 2007; Shannon et al., 2013) and primates (Gilmer & McKinney, 2003). The limited space available to groups of captive cetaceans may also impact their ability to use dispersal to keep intra-group aggression to a minimum. In this regard, preliminary evidence suggests that conflict management in the form of post-conflict affiliation in captive cetaceans may be more frequent than in wild counterparts because captive animals cannot disperse to prevent the resumption of aggression (Yamamoto et al., 2015; Sánchez-Hernández et al., 2019). More research on this topic is needed.

### Feeding and cognitive demands

Feeding is not just about fulfilling dietary requirements; it also encompasses vital cognitive and physical challenges that are essential to well-being. Consistent with the notion that the cognitive demands of feeding are integral parts of welfare, Clegg et al. (2023) found that, when captive bottlenose dolphins are presented with cognitively enriched foraging opportunities, they exhibit more positive (*e.g.*, greater engagement and healthy appetite) and fewer negative (*e.g.*, stereotypies) welfare behaviors.

Dietary preferences and needs vary widely across cetacean species and across communities within species. For instance, southern resident orcas in the Pacific Northwest have a diet that consists of nearly 80% Chinook salmon, depending on the season. The emphasis on Chinook is not just a preference; it is a vital aspect of their culture (Hanson et al., 2021). Orcas off the coast of New Zealand, on the other hand, specialize in eating stingrays, requiring a high level of skill in processing before they are ingested because of the danger of stingray spine penetrations (Duignan et al., 2000). Orcas can also eat highly varied diets, sometimes including mammals, sea birds, and fish (Samarra et al., 2018). Other species, like common bottlenose dolphins, also have quite diverse diets (Gannon & Waples, 2004). In contrast, captive cetaceans are fed a narrower selection of commercially available dead fish and occasional invertebrates (Rosen & Worthy, 2018). Rosen & Worthy (2018, p. 719) note “both a lack of diet diversity and the reliance on frozen foods present potential nutritional challenges”. Although the fish are generally of high quality (*i.e.,* freshly frozen and thawed, free from contaminants, regularly tested, and meeting balanced nutritional requirements), the freezing and thawing of fish results in significant nutrient loss; to replenish these nutrients and avoid dietary disease, supplements (*e.g.*, thiamine, vitamin E) are often supplied (Brando et al., 2018). One of the reasons for providing a limited selection and uniform delivery is to maintain records of how much each individual is eating, as appetite is an important health measure.

Fish are delivered to captive cetaceans in a manner (*i.e.,* thrown directly into their mouths above water) that requires little to none of the cognitive activity involved in natural hunting and feeding. There does appear to be some enrichment benefit for the animals when food is delivered as part of cognitive challenges during positive reinforcement training (Jaakkola, 2024). The lack of stimulation from the way food is delivered must be countered by the implementation of other methods of cognitive enrichment.

It must be understood that animals are evolutionarily driven to seek the most accessible and abundant food resources for survival. However, the routine consumption of readily available food sources, such as occurs in fish provisioning, ecotourism, or in the captive setting, does not satisfy the biological drive to engage complex cognitive and physical faculties inherent in foraging and hunting, which involves travelling to locate food along with capturing and consuming the prey. These activities present diverse opportunities and cognitive challenges that constitute a large percentage of cetaceans’ time budget in the wild (Neumann, 2001; Stockin et al., 2009; Noren & Hauser, 2016).

### Interactions with humans

Captive cetaceans are often required to interact with humans in different ways, for husbandry, training, and entertainment of the public—all of which have an effect on welfare. The Human-Animal Relationship is recognized within the Five Domains model of animal welfare (includes: nutrition, environment, health, behaviour, and mental state; Mellor et al., 2020). Such interactions are known to affect welfare in both positive (*i.e.,* positive affective engagement) and negative ways (*e.g.*, demotivating negative affect; Mellor, 2015; Mellor et al., 2020). Training for interactions with the public, animal husbandry, and veterinary care is conducted through positive reinforcement methods whereby the animals are rewarded with something they like, typically food, for performing a requested behavior. It has been suggested that training and performance are a form of environmental enrichment for captive cetaceans (Westlund, 2014; Jaakkola et al., 2023; Melfi & Ward, 2020). Training sessions for dolphins in accredited facilities are also associated with positive welfare indicators (*e.g.*, play behavior and behavioral diversity; Miller et al., 2021c). Positive reinforcement training in accredited facilities has been shown to help decrease stereotypic behavior and stress in harbor porpoises (Desportes et al., 2007) and other species (rhesus macaques: Coleman & Maier, 2010; chimpanzees: Pomerantz & Terkel, 2009). Training is considered to be enriching insofar as it provides the animal with opportunities to learn (Melfi, 2013) and provides welfare benefits that extend beyond the training session itself (Fernandez, 2022). There is also evidence to suggest that dolphins exhibit anticipatory behavior (*e.g.*, surface look, spy hop) prior to interacting with their trainers or receiving toys, suggesting anticipatory behavior as a measure of motivation (Clegg & Delfour, 2018; Clegg et al., 2018). In this regard, bottlenose dolphins have been shown to change the structural characteristics of signature whistles in association with staff presence and food-anticipatory activity, indicating increased arousal (Probert et al., 2021). Nevertheless, more research is required to understand which components of training and performance are indeed enriching and long-lasting rather than just providing temporary relief from boredom, creating a distraction, or otherwise occupying the time budget of captive cetaceans.

Standards that safeguard animal well-being in interactions with visitors (*e.g.*, dolphin assisted therapy and other swim programs, petting pools, and public feeding activities; Stewart & Marino, 2009) vary. In many respects, standards implemented by the US Animal Welfare Act (2023), Alliance for Marine Mammal Parks Aquariums (AMMPA) (2025), Association of Zoos & Aquariums (AZA) (2025), and EAAM (2019) mirror one another. However, an important difference is found in standards for interaction time. The Animal Welfare Act (2023) requires that interaction time (*i.e.,* designated interactive swim sessions) for each cetacean cannot exceed two hours per day, with at least one period of at least 10 continuous hours without public interaction within a 24-hour period. AMMPA, EAAM and AZA standards do not require a similar 2-hour cap. The Alliance for Marine Mammal Parks Aquariums (AMMPA) (2025) requires that each cetacean have at least one period of at least 10 continuous hours without public interaction in each 24 h period, and EAAM (2019) standards require one period of at least 12 continuous hours without public interaction within a 24-hour period. Under AMMPA and EAAM standards, a cetacean can be exposed to extended hours of interaction time. The Association of Zoos & Aquariums (AZA) (2025) standards require that certain staff determine interaction time based on various factors. Another difference is in the ratio of human participants to cetaceans. The Animal Welfare Act (2023) states that the ratio of human participants to cetaceans shall not exceed 3:1. The Alliance for Marine Mammal Parks Aquariums (AMMPA) (2025), Association of Zoos & Aquariums (AZA) (2025), and EAAM (2019) do not provide a specific ratio; their standards require that the ratio of human participants to cetaceans should be appropriate to the type of interactive activity offered, although they require approval of the ratio by certain staff.

Several factors may play an important role in stress levels during interactive programs and therefore the issue is very complicated. Matsushiro et al. (2021) studied only five dolphins, showing that the average cortisol level of the group decreased significantly after an interaction session. But average cortisol levels were significantly higher in the busy visitor season than in low visitor season in two out of three dolphins. They concluded that there was little evidence that interactions caused acute stress but kept open the possibility of chronic stress in dolphins participating in interactive programs during the high visitor season. It should also be noted that cortisol is not necessarily harmful unless it becomes dysregulated during periods of chronic stress (Sapolsky, Romero & Munck, 2000; Jacobs et al., 2021). One study in New Zealand, with a small sample size of three dolphins, found that common dolphins significantly increased their use of refuge areas when exposed to the public in SWD attractions (Kyngdon, Minot & Stafford, 2003), suggesting these sessions may be aversive or stressful. However, they found no overall decrease in welfare due to SWD activities. Miller et al. (2011), however, found higher rates of behavioral diversity following swim programs, which has been interpreted as a positive welfare indicator (Miller et al., 2020; Brereton & Fernandez, 2022).

More changes in behavior have been reported during unstructured or free-style SWD sessions compared to structured (staged) sessions in which there is explicit trainer regulation of interactions between dolphins and human swimmers (Brensing et al., 2005). Brando, Kooistra & Hosey (2019) found that some dolphin behaviors change during swim sessions but that these changes may be due to the presence or absence of trainers or disturbances in the pool rather than due to the swim session itself. Given the available data, it is possible that structured programs are more positive for captive cetaceans than unstructured sessions. But there are many dimensions to interactions between humans and captive cetaceans that still need to be analyzed. Delfour et al. (2020) developed a method of analyzing dolphin-trainer interactions during training sessions that may have promising applicability to SWD sessions but is not yet fully validated as a direct measure of welfare. More research on important physiological and behavioral factors (*i.e.,* oxytocin levels, more precise cortisol levels) may elucidate the nature of the dolphin-trainer relationship and how it impacts dolphin welfare.

## Current Welfare Issues

The welfare of cetaceans, as with any animal, is multidimensional; each factor involved in their overall well-being is intricately linked to several others. A deep understanding of captive cetacean welfare requires appreciating that many welfare problems, particularly health challenges that present a risk to welfare (*e.g.*, infections, parasites, etc.) occur both in captivity and in the wild. But the critical point is that there are numerous known reasons for illness, injury, and mortality in the wild, including pollution, pathogens, predators, ships, noise, nets, and so on (Bossart et al., 2003; Fair et al., 2017; Avila, Kaschner & Dormann, 2018; Bossart et al., 2019; Sanganyado & Liu, 2022). None of these have any relevance to land based tanks, leaving open the possibility that there are other factors (*e.g.*, stress, novel pathogen and chemical exposure, neurobiological harm) unique to or exacerbated by captivity that contribute to poor welfare and death in captive cetaceans. For example, below we present evidence suggesting that at least some common diseases of captive cetaceans are directly linked to husbandry or environmental conditions. Despite decades of housing cetaceans in captivity and efforts by accredited zoos and aquaria to modify their facilities, husbandry, and preventative medical programs to reduce these diseases, many of them persist.

Although sufficient evidence exists to raise concerns about the health and welfare of captive cetaceans, as is detailed in the present paper, it is important to note that a great deal of data on captive cetacean disease and illness is absent from the literature and is publicly inaccessible. The reason for this is that the medical and behavioral records of these animals are routinely withheld by the institutions housing them, including cause of death data and necropsy reports for deceased captive cetaceans. Despite the value of such records for advancing captive cetacean health, husbandry, and welfare, as well as wildlife conservation efforts, federal agencies with jurisdiction over these animals and facilities have routinely failed to enforce their authority to obtain such records (Rally, Baur & McFeele, 2018). Therefore, it can be expected that the information available and presented herein is likely an under-representation of the true rates and etiologies of morbidity in captive populations.

In this section, we describe the current well-being (mental and physical health) of captive cetaceans, starting with brain and behavioral issues, various dimensions of physical health, and concluding with longevity, survival, and mortality statistics. The chronic stress of coping with the various dimensions of captivity over time has an impact on mental and physical health in cetaceans, as it does in every other species (Marino et al., 2020). All these factors, described more fully below, contribute to a generally problematic picture of captive cetacean welfare.

### Brain and Behavior

#### Stereotypies

One of the more prevalent behavioral abnormalities found in captive animals is stereotypic behavior (Mason & Latham, 2004; Mason & Rushen, 2008; Bacon, 2018). When present, stereotypies reflect changes in the brain. The circuitry involved in motor control and stereotypies is complex but, at the neural center of this circuitry, is the basal ganglia (or corpus striatum), which are highly conserved across mammals, including cetaceans (Grillner & Robertson, 2016; Marino, 2022). Therefore, cetacean stereotypies, like stereotypies in other mammals, are not just behavioral issues, but also represent brain function abnormalities (Jacobs et al., 2021).

Captive cetaceans exhibit a range of stereotypies that are also found in other captive species (Clubb & Mason, 2003; Morgan & Tromborg, 2007; Mason, 2010) but not found in free-living cetaceans. Although much more in-depth research is needed on the frequency and nature of stereotypies in captive cetaceans (Gygax, 1993; Clark, 2013), oral stereotypies appear to be relatively common (Jett et al., 2017). These are observed most frequently, but not exclusively, in captive orcas and include biting, chewing, and jaw-popping on hard tank surfaces (Ventre & Jett, 2015; Visser & Lisker, 2016; Jett et al., 2017). Also found in captive cetaceans are circling and repetitive swimming patterns (Ugaz et al., 2013). However, in the Cetacean Welfare Study, Miller et al. (2021a) did not find route tracing behavior (a form of stereotypical swimming) in the captive dolphins they studied but rather a high rate of behavioral diversity (amount of species typical behavior), suggesting an inverse relationship between behavioral diversity and stereotypies. This is consistent with Clegg et al. (2023), who found that stereotypic behavior accounted for only 0.01% of the activity budget in dolphins receiving cognitive enrichment at Kolmårdens Djurpark. Continued research is necessary to validate behavioral diversity as an indicator of positive welfare for bottlenose dolphins and other captive cetaceans (Miller et al., 2020; Brereton & Fernandez, 2022).

Self-harm, whether deliberate or incidental to other abnormal behaviors like stereotypies, also occurs in captive cetaceans but, again, is largely unobserved in free-living cetaceans (although these kinds of behaviors are more difficult to observe in the wild). Examples include constantly rubbing their skin on hard objects, resulting in excessive abrasions (Lipman, 2016), or continually hitting against the side of the tank (Dima & Gache, 2004). In one well-known case, a captive orca continually hit his head against the sides of the tank until he died of a brain aneurysm in 1980 (Ringelestein, 2021). Repetitive regurgitation and reingestion is another stereotypic behavior that develops in response to boredom, illness, social isolation/instability, and/or stressful training interactions (Walsh et al., 1996; Calle, 2005). On the other end of the activity spectrum are symptoms that may indicate depression (*e.g.*, spending more time logging or lying motionless on the surface of the water for extended periods (Jett & Ventre, 2011), resting motionless on the bottom of the tank, and loss of appetite in the absence of physical illness (Dima & Gache, 2004; Jett & Ventre, 2012; Worthy et al., 2013). It should be noted that lying on the bottom of the tank is sometimes observed in resting individuals who are not necessarily depressed. These various factors are best interpreted in a more holistic way, such as assessing whether many different indicators of depression are present rather than just one. Captive facilities attempt to control or diminish stereotypies primarily through reinforcement of alternative behaviors, pharmacological treatment, or environmental enrichment, but none are completely successful (Mason et al., 2007). One study did find that cognitive foraging enrichment led to reduced stereotypies and other abnormal behaviors (Clegg et al., 2023), suggesting that more complex enrichment formats anchored in more naturalistic behaviors may be more helpful than non-cognitive forms.

#### Aggression

Although aggression towards members of one’s family or social group does occur in free-living cetaceans (Scott et al., 2005; Marley, Cheney & Thompson, 2013; Robinson, 2014), it is often kept from escalating by dispersal and other factors (Bisther, 2002; Towers et al., 2018). But conspecific aggression can sometimes be exacerbated in tanks, where space is inadequate for dispersal, is shared by individuals who are not necessarily compatible, and where separation or isolation might be the only recourse—all of which have welfare ramifications (Frohoff, 2004; Evans, 2015; Ventre & Jett, 2015). Miller et al. (2021c) suggest that for species with dominance hierarchies, such as bottlenose dolphins, the ability to have the space to separate themselves physically from other individuals may be important for welfare. Likewise, direct attacks on humans by orcas and belugas in the wild are unknown (Pagel, Scheer & Lück, 2017). There is one early report of a dangerous encounter between a woman and a pilot whale (Shane, Tepley & Costello, 1993) and one record of a free-living bottlenose dolphin killing a human (who had been abusing the dolphin; Santos, 1997). Nevertheless, there have been hundreds of aggressive acts by captive cetaceans towards humans (Lott & Williamson, 2017), and four human deaths by captive orcas (Parsons, 2012). There is, in fact, a long record of aggression by captive orcas, many in petting/interaction pools, towards humans, resulting in severe injuries (Anderson, Waayers & Knight, 2016).

### Physical health

#### Nutrition and metabolism

Many diseases in captive cetaceans are associated with metabolic syndromes believed to be linked to the captive diet (Rosen & Worthy, 2018). Captive bottlenose dolphins, for example, are prone to developing insulin resistance and fatty liver disease, similar to type-2 diabetes in humans (Wells et al., 2013; Colegrove, 2018). Free-living bottlenose dolphins have been found to be 15 times more likely to express lower iron levels than captive dolphins (Mazzaro et al., 2012). Elevated iron found in captive bottlenose dolphins as they age is a precursor to developing a disease called hemochromatosis, which is known to occur in managed care populations. This suggests that captive dolphins are more susceptible to non-hereditary hemochromatosis than free-living populations, which can lead to liver, heart, and reproductive problems, as well as joint pain, increased cancer risks, and death (Mazzaro et al., 2012; Venn-Watson et al., 2012a; Venn-Watson et al., 2013b). Another condition likely related to the captive diet is hypocitraturia, which is characterized by low levels of citrate urinary excretion (Zuckerman & Assimos, 2009). This disease is four times more common in captive than free-living bottlenose dolphins and promotes the formation of kidney stones, which can lead to serious complications such as renal failure and death (Venn-Watson et al., 2010).

#### Skin health

Skin disease is common in captive dolphins, with the most prevalent being tattoo skin disease (TSD; black or grey stippling discoloration of the skin), caused by poxvirus, and diamond skin disease (slightly raised, rhomboidal grey patches), caused by the bacterium *Erysipelothrix rhusiopathia*. Skin diseases are also found in dolphins in the wild; for example TSD is associated with poor population health (Van Bressem et al., 2008). Although poxvirus is associated with TSD lesions in bottlenose dolphins, the virus typically is expressed in individuals who are immunocompromised from stress or concurrent illness. Van Bressem et al. (2008) reported that 20.6% of the 257 bottlenose dolphins held in 31 US and European facilities had tattoo lesions. When left untreated, active diamond skin disease lesions can progress to a serious and life-threatening zoonotic bacterial infection (Tryland, 2018; Lacave et al., 2019). However, it is also possible for cetaceans to develop sudden illness and death in the absence of obvious skin lesions. The causative organism is found on dead fish products, the main source of infection for captive cetaceans (Lacave et al., 2019). For this reason, Erysipelas is primarily a disease of captive cetaceans (Rosen & Worthy, 2018) and numerous deaths have been reported due to peracute septicemic *Erysipelothrix* infection (Van Bonn et al., 2007).

#### Dental disease

Tooth injury resulting from stereotypic behavior is a major problem for many animals across a range of species in zoo settings (Glatt, Francl & Scheels, 2008). Mason & Latham (2004) estimated that 82% of wild carnivores held in zoos express stereotypical behavior, with oral stereotypies being most prevalent (Bergeron et al., 2006). Tooth wear from oral stereotypies is also commonly reported in captive orcas (Graham & Dow, 1990; Jett & Ventre, 2012; Ventre & Jett, 2015; Almunia, 2017). Even though they are fed in a way that does not involve using their teeth (by throwing fish into the back of the throat), captive orca teeth commonly exhibit extensive wear and other dental pathologies such as fractures and exposed pulp cavities (Jett & Ventre, 2012; Ventre & Jett, 2015; Visser & Lisker, 2016; Jett et al., 2017). The main reason for the extensive dental wear and trauma in captive orcas is frequent biting and grating of the teeth against hard surfaces in the tank. This stress-related stereotypy does not appear to be as problematic in captive belugas and bottlenose dolphins as in orcas. In one large survey, approximately 69% of captive orcas in the US and Spain had fractured mandibular teeth and 24% exhibited “major” to “extreme” mandibular coronal tooth wear down to the gingiva (Jett et al., 2017).

Natural tooth wear is associated with increasing age in free-living odontocetes (Perrin, Myrick Jr & International Whaling Commission, 1980; Ramos, Di Beneditto & Lima, 2000; Foote et al., 2009; Loch & Simões Lopes, 2013; Loch et al., 2025); however, advanced tooth damage from traumatic crown injury is rare in free-living orcas (Loch et al., 2025). Moreover, tooth wear in orcas in the wild is related to specific ways some orca subtypes feed. For example, when evaluating the dentition of three orca ecotypes (*i.e.,* Offshore, Transient, and Resident), Ford et al. (2011) suggested that the abrasive skin of sleeper sharks (*Somniosus pacificus*), a frequent prey of Offshore orcas but not the other two subtypes, was implicated in their pronounced dental wear.

When teeth are gradually traumatized over the lifetime of an animal, such as on the rough skin of prey or from water and tongue movements during suction feeding (Marx et al., 2023), internal healing mechanisms are initiated to protect the integrity of the pulp chamber, such as tertiary dentin formation (Loch et al., 2025). The superficial forms of dental wear commonly seen in free-ranging orca (*e.g.*, attrition, abrasion, abfraction, and erosion) are gradual enough to allow for concurrent healing processes and do not result in pulp exposure and subsequent infection (Loch et al., 2025). However, in captivity, cetaceans experience different types of dental injury such as acute onset of traumatic complicated crown fractures that do pose a significant health threat, including pulp infection, tooth root abscess formation, and bacteremia (Holmstrom, 2018).

In an effort to avoid infection and health complications, captive orcas with damaged teeth are often forced to undergo a modified pulpotomy procedure whereby the teeth are drilled, and pulp and debris are removed (Ventre & Jett, 2015). To mitigate the risk of systemic infection, damaged teeth are thereafter flushed with antiseptic solutions daily and the animals are often routinely treated with antibiotics, which may result in drug-resistant pathogens (Davies & Davies, 2010; Dold, 2015) and altered immune system function (Yang et al., 2017).

#### Digestive and gastrointestinal disease

Diseases of the gastrointestinal tract, such as gastritis and gastric ulceration, have been seen in both free-living and captive cetaceans. Gastric ulcers are one of the most common gastrointestinal diseases in captive cetaceans (Colegrove, 2018). In many cases, their etiology remains unclear; however, ulcers in captive cetaceans have been linked (or suspected to be linked) to social stress (Joseph, Ollermann & Gridley, 2019), dietary factors and food quality (Geraci & Gerstmann, 1966; Rosen & Worthy, 2018), foreign body ingestion (Buhrmann, Gridley & Oellermann, 2023), and the administration of non-steroidal anti-inflammatory drugs (Simeone et al., 2014), as well as the bacteria *Helicobacter delphinicola* (Segawa et al., 2023). A recent study of this bacterial organism in dolphinaria in Japan found a statistically significant relationship between the presence of this organism and chronic gastrointestinal disease in captive bottlenose dolphins and found evidence to suggest that transmission occurred rapidly between individuals sharing the same pool enclosure (Segawa et al., 2023). *Helicobacter* gastritis and ulceration can lead to perforation of the stomach lining (Stoskopf, 2015) and has been associated with deadly cases of stomach torsion (Begeman et al., 2013).

Also, repetitive regurgitation and reingestion can result in chronic irritation of the delicate mucosal surfaces of the esophagus from routine exposure to acidic stomach contents, leading to inflammation, corrosion, and even death (Walsh et al., 1996). This behavior, as opposed to simple regurgitation or vomiting, has not been reported in free-living cetaceans. Intestinal volvulus (an abnormal rotation of the intestine), another deadly gastrointestinal disease found in free-living and captive cetaceans, is a common cause of mortality in both (Begeman et al., 2013). Although the etiology of this condition remains unclear in most reported cetacean deaths, a significant factor known to influence the development of intestinal torsion and volvulus in a wide array of other mammalian species is dysbiosis, or an imbalance in the natural gut microbiota (Weese et al., 2015; Hullar et al., 2018; Oliveira et al., 2024). In a variety of non-human animals, and in humans, there appears to be a strong relationship between disturbances in the gut microbiota and chronic stress and depression (Kelly et al., 2016; Peirce & Alviña, 2019; Yang et al., 2020). Additional studies are necessary to understand the impact of the various social, environmental, and husbandry factors influencing the cetacean intestinal microbiome and how these might also affect mental health. However, numerous studies have shown a clear and significant impact of captivity on the fecal microflora of captive cetaceans (Bai et al., 2021; Suzuki et al., 2021; Shah et al., 2024), which consequently impacts both gastrointestinal health and the systemic immune system (Linnehan et al., 2024).

#### Infectious disease

Viral, bacterial, and fungal infections are found in both captive and free-living cetaceans, with viral and bacterial pneumonia the most common causes of fatality in captive cetaceans (Kielty, 2011; Jett & Ventre, 2012; Nelson et al., 2019). Pulmonary mycotic infection (fungal pneumonia) is also a frequent cause of death in captive and free-living cetaceans (Brando et al., 2018; Reidarson, García-Párraga & Wiederhold, 2018). At least 15 of the 22 orcas who died in US marine parks between 1990 and 2010 succumbed to infectious and inflammatory diseases, including pneumonia, encephalitis, bacteremia, and leptomeningitis (Kielty, 2011). In a retrospective study of the US Navy Marine Mammal Program from 1980–2010, 50% of the dolphins had histopathologically confirmed pneumonia (Venn-Watson, Daniels & Smith, 2012b). Other infectious diseases in captive cetaceans are related to behavioural dysfunctions. For instance, it has been suggested that the increased time orcas spend floating on the tank surface increases vulnerability to mosquito-borne infections, such as St. Louis encephalitis (Jett & Ventre, 2012). At least two captive orcas deaths from this disease have been documented (St. Leger et al., 2011; Jett & Ventre, 2012).

Routine preventative administration of antibiotics and antifungals often causes an imbalance of microflora and resistance to the medicines themselves (Dold, 2015; Reidarson, García-Párraga & Wiederhold, 2018; Park et al., 2020). For instance, meticillin-resistant *Staphylococcus aureus* (MRSA) was reported in captive dolphins in two Italian facilities, with one dolphin in each facility dying from MRSA-linked septicemia (Gili, Biancani & Mazzariol, 2017). Another recent case demonstrated the presence of resistant *Morganella morganii*, a bacterium associated with fatal sepsis in human beings, in a captive bottlenose dolphin in South Korea (Park et al., 2020). *Candidiasis*, often observed in immunocompromised individuals, is increasingly prevalent in captive cetaceans (Reidarson, García-Párraga & Wiederhold, 2018; Ohno et al., 2019). Several other opportunistic infections (*e.g.*, *Giardia* sp. and *Cryptosporidium* spp.) have also been linked to the captive environment (Koch et al., 2018).

#### Reproduction

Most cases of neonatal death in captive facilities occur in young females giving birth for the first time (Owen, 1990; Sweeney et al., 2010), which may be due to immaturity and lack of exposure to natural mother-calf relationships (*i.e.,* factors particularly relevant to the captive situation). The same pattern is true in the wild (Henderson et al., 2014; Robinson et al., 2017; Wells et al., 2025). However, the fact that these infant mortality statistics are the same in the wild and in captivity—where none of the dangers or risks inherent in the wild are present—leaves open the critical question of why neonatal deaths in captivity are not lower than in the wild.

Female dolphins at SeaWorld facilities are often impregnated through artificial insemination (AI), an invasive procedure that requires mild sedation so that semen can be deposited inside the reproductive tract through a catheter (Robeck et al., 2005a; Robeck et al., 2005b; O’Brien & Robeck, 2006). AI often involves multiple attempts. In the common bottlenose dolphin, the conception rate after AI using frozen–thawed semen is 65–70% (Robeck, O’Brien & Odell, 2009). SeaWorld also employs AI to control the sex ratio of its captive dolphin population in favor of breeding females. Out of 30 such inseminations, 28 have resulted in females (Robeck & O’Brien, 2018).

Orca females reach sexual maturity between 11 and 13 years of age in the wild (Ford, 2018) but reach peak fertility at around 20 years of age (Ward, Holmes & Balcomb, 2009). Females typically give birth to their first viable calf at 12–14 years of age (Olesiuk, Ellis & Ford, 2005). Calves nurse exclusively for at least a year but remain in close association with their mother for at least the first two years or longer (NOAA Fisheries, 2025). Males sexually mature at the age of 15, but do not typically reproduce until age 21 (Ford, 2009). AI is used to breed captive orcas as well and several have been conceived at SeaWorld parks this way (Robeck et al., 2004; Robeck, Steinman & O’Brien, 2017).

Female beluga whales in the wild become sexually mature at age 8–12 years, males between 9–15 years—although there is still considerable variability in estimates (O’Corry-Crowe, 2009; Suydam, 2009). Most calves continue nursing until they are 20 months old, although nursing is often available to calves for more than two years (O’Corry-Crowe, 2009). Captive beluga reproduction has been a longstanding problem because of physiological and behavioral factors (*e.g.*, they are facultative induced ovulators and seasonal breeders; Steinman et al., 2012). That is, they are not always ready to breed. Therefore, AI has been used, with uneven success, in captive beluga whales (O’Brien et al., 2008; Robeck et al., 2010).

#### Longevity, survival, and mortality rates

There is variation in estimates of life expectancy for free-living bottlenose dolphins for a large variety of reasons (*e.g.*, distinct wild populations with different health challenges, variation across managed facilities; Jaakkola & Willis, 2019). An earlier estimate for free-living bottlenose dolphins suggested a lifespan of ∼25 years (Sergeant, Caldwell & Caldwell, 1973). Wells & Scott (2009) suggest that free-living female bottlenose dolphins can live to more than 56 years and males to 48 years. Wells et al. (2013) suggest an average age at death (which is different from maximum lifespan) of 19.9 years. For captive dolphins, the managed population studied by Venn-Watson et al. (2013a) had an age at death of 32.6 years. More recent research on captive dolphins in US zoological facilities suggests a mean life expectancy of 28.2 years, which is significantly higher than it is for the two out of three free-living populations used for comparison by Jaakkola & Willis (2019). The most recent study of longevity in zoological facilities also indicates improvements in life expectancy for captive bottlenose dolphins (longer than wild counterparts) and in first year survival rates (Tidière et al., 2023). These increases were attributed to improvements in captive environments and to the fact that there were fewer wild-caught dolphins, who have higher mortality rates, in the more recent time periods examined. Similarly, although high rates of neonatal mortality have historically been considered a major problem in captivity (Van Lint et al., 2006), there have been improvements in the percentage of captive bottlenose dolphin calves living past the first year in accredited facilities (Sweeney et al., 2010). One crucial point remains: Tidière et al. (2023, p. 7) acknowledge that their study “does not assess individual-level welfare or quality of life, which is essential to advance animal care and develop a holistic understanding of animal welfare”. Thus, although longevity for common bottlenose dolphins appears to have increased over time in captive settings, it is important to remember that longevity is not synonymous with quality of life.

For orcas, the mean life expectancy for females in the Pacific Northwest is ∼46–50 years, with a maximum of ∼80 years (Olesiuk, Ellis & Ford, 2005; Ford, 2018). Mean life expectancy for males is ∼30 years but they may live to ∼60 years (Ford, 2018). In captivity, calf mortality rates within the first six months are similar to what is seen in the wild, but the majority of captive orcas do not survive to the age of thirty (Jett & Ventre, 2015). Jett & Ventre (2015) found that orcas in US facilities (12.0 years) demonstrated a significantly higher median survival time than those in non-US facilities (4.4 years), as did whales entering captivity after January 1985 (11.8 years) *versus* those entering prior to January 1985 (3.9 years). Although Robeck et al. (2015) estimated life expectancy in captive orcas to be 47.7 years, there are multiple controversies about this estimate (Jett & Ventre, 2015; Callaway, 2016; Franks et al., 2016; Jett, 2016; Robeck et al., 2016a; Robeck et al., 2016b). As such, despite the findings that survival of captive orcas has generally increased in the last four decades, survival to age milestones remain poor in captive orcas when compared to free-living orcas.

There is considerable variation in mean life expectancy for beluga whales. Willis (2012) suggested a broad range of ∼12–66 years. A recent review of beluga age estimation based on dentinal growth layer group supports a maximum longevity of 30–35 years in free-ranging animals (Brodie, Ramirez & Haulena, 2013). Also based on dental measures, Luque & Ferguson (2010) provided a maximum age estimate at ∼75–80 years. Although definitive life expectancy statistics for captive beluga whales have not yet been established, there is evidence that lifespan is compromised (Woodley, Hannah & Lavigne, 1997). The best estimation for maximum longevity in captive beluga whales is 35 years of age (Montano et al., 2017). In older studies, survival rates in captive belugas also appeared to be lower than in nature (Small & De Master, 1995; Woodley, Hannah & Lavigne, 1997). Robeck et al. (2005a) and Robeck et al. (2005b) found that only 14 out of 22 beluga births (63.6%) in captive facilities resulted in calves that reach two years of age or older. However, updated statistics for captive and various populations of free-living belugas are clearly needed.

Although more data are needed, evidence shows that captive bottlenose dolphin survival rates are at least as high as those for certain wild populations (Jaakkola & Willis, 2019). However, survival rates for captive belugas and orcas remain relatively poor (Robeck et al., 2005a; Robeck et al., 2005b; Jett & Ventre, 2015). Cetaceans in captivity are afforded full-time veterinary care and are protected from food shortages, predators, pollution, and parasites, suggesting they should be living longer than those in the wild and potentially reaching maximum lifespan. This situation begs the question of why survival rates are not higher for captive cetaceans, and highlights the need for more research in this area.

## Environmental Enrichment

Whether zoos and marine parks can, in principle, provide for the needs of cetaceans is a question that has received increasing attention in the domain of environmental enrichment (Brando et al., 2018; Jacobs et al., 2021). The marine park industry was the first to recognize the difficulties cetaceans have in coping with the incongruity between artificial and natural environments (Swaisgood & Shepherdson, 2005; Morgan & Tromborg, 2007). As a result, accrediting agencies require various forms of enrichment, which are implemented to “maximize psychological health” (WAZA, 2025), to stimulate “natural behavior” (Association of Zoos & Aquariums (AZA), 2025), to allow the animal “variety and choices” in their environment (Alliance for Marine Mammal Parks Aquariums (AMMPA), 2025), and to provide the animal with “behavioural choices” and “control over its environment” (EAAM, 2019). The requirement of such ad hoc enrichment constitutes a de facto recognition that the captive environment is inherently impoverished, as has been suggested by several researchers (Hancocks, 2002; Mason & Burn, 2018; Jacobs et al., 2021). This claim has been refuted for accredited dolphin enclosures by Jaakkola (2023), but Jaakkola does not consider facilities housing larger cetaceans or those that are unaccredited. Nevertheless, it should be noted that this refutation is based on an oversimplified interpretation of the impoverishment-enrichment continuum used in laboratory studies whereby Jaakkola isomorphically maps the laboratory paradigm onto captive dolphin enclosures. Simply adding enrichment to an enclosure does not necessarily transition an impoverished environment to an enriched environment (Jacobs et al., 2021).

Traditionally, much of the enrichment for captive cetaceans has involved the presentation of plastic or rubber floating objects (*i.e.,* toys) with which the animals can interact (Brando et al., 2018; Lauderdale et al., 2021e; Jaakkola, 2024). Although such objects may initially arouse interest, habituation occurs relatively quickly (Clark, 2013), which is why variable enrichment schedules and novel items are recommended (Kuczaj et al., 2002). It nevertheless remains unclear to what extent such objects are actually enriching (Delfour & Beyer, 2012). Other types of enrichment may include submerged objects, human interaction/training, as well as food-based, structural, and sensory enrichment (Brando et al., 2018), including classical music (Guérineau et al., 2022). More recent efforts have focused on adding cognitive challenges (*e.g.*, physical or virtual puzzles and games) to the enrichment repertoire (Clark, 2013; Jaakkola, 2024). As summarized by Jaakkola (2024), cognitive stimulation appears to be intrinsically rewarding and has been associated with a variety of positive welfare indicators (*e.g.*, increases in activity, decreased stereotypies, increased exploratory behavior). Yeater et al. (2024) taught dolphins the concept of innovation and found that the activity was intrinsically rewarding and cognitively engaging. Clegg, Borgner-Turner & Eskelinen (2015) found that cognitive foraging enrichment improved welfare by increasing dolphin engagement and motivation in training sessions and led to fewer stereotypic behaviors. Such cognitive challenges need to account for both species and individual differences and need to be at an appropriate level of difficulty to avoid negative welfare outcomes (*e.g.*, unresolved frustration; Jaakkola, 2024). Unfortunately, enrichment activities that are time-consuming tend to be implemented less frequently (Hoy, Murray & Tribe, 2010). Moreover, as of 2017, cognitive enrichment appeared to be the least-used type of enrichment in captive settings (Clark, 2017). However, cognitive-focused enrichment practices have grown in recent years and appear to be more effective in diminishing abnormal stress-related behaviors, at least for smaller cetaceans (Perlado-Campos, 2017; Matrai et al., 2020).

Neuroscience research suggests that a more natural environment is better for the brain and for the emotional health of the animal than are artificially enriched environments (Bennett, Diamond & Rosenzweig, 1972; Lambert et al., 2015; Lambert et al., 2016). Consistent with this notion are studies that suggest that dolphins in captive environments with more natural elements (*e.g.*, sea pens or netted off areas continuous with the ocean) are less stressed and display fewer behavioral abnormalities than those living in tanks. For instance, Ugaz, Sánchez & Galindo (2009) found that the same group of bottlenose dolphins engaged in more active swimming and less logging in open sea pens (with access to ocean and a more complex environment) than when they were in closed facilities with no ocean access. As noted previously, dolphins in closed artificial environments were also found to have higher salivary cortisol levels than those in open sea pens (Ugaz et al., 2013). Moreover, dolphins transferred from concrete tanks to captive ocean environments spent less time in social interactions (Ruiz, Sánchez & Maldonado, 2009). The authors suggested this finding could be due to the significant increase in space available (the ocean habitat was approximately five times the size of the tanks) as well as the opportunity to explore other features of the natural environment, such as fish and other organisms who may be present (Ruiz, Sánchez & Maldonado, 2009).

The above studies, while far from conclusive, strongly suggest that there are welfare benefits to dolphins (and likely other cetaceans) of living in a more natural ocean environment—even when captive. The current literature on environmental enrichment and welfare in cetaceans indicates that much more research is needed to determine the extent to which specific enrichment efforts can improve welfare, with the caveat that certain aspects of enrichment (*e.g.*, space) often cannot reasonably be addressed in traditional captive facilities. The effects of environmental enrichment can only be fully understood by employing well-confirmed welfare tests (Brereton & Rose, 2023).

## Discussion and Conclusion

In the present review, to summarize, we discussed some of the most relevant and substantive aspects of captive environments for cetaceans. These include the amount of space provided but also the complexity of the environment. Recent studies suggest that the complexity of a captive environment, and not just the size, is important. The nature of sensory-perceptual experience in captivity is also important for welfare and, as cetaceans are highly acoustically sensitive animals, more research is needed to understand how tank size and shape affects them in terms of acoustic behavior. Other relevant factors are sociality and how groupings in captivity do or do not resemble natural social groups and the longstanding effects on welfare. Finally, feeding, cognitive demands, and interactions with humans are discussed in terms of whether food is not only balanced and nutritional but also is presented in a stimulating way. We then described the mental and physical health of captive cetaceans, starting with brain and behavioral issues, various dimensions of physical health, and concluding with longevity, survival, and mortality statistics. Captive cetaceans, and especially orcas, exhibit several abnormal behaviors in captivity, including oral stereotypies, self harm, and hyper-aggression towards tankmates and humans. In terms of physical health, captive cetaceans suffer from all of the systemic, skin, and digestive diseases known to occur in cetaceans in the wild. This finding begs the question of why they are exhibiting these diseases in a clean, controlled environment. Moreover, reproduction and births remain problematic in captivity. Finally, with the exception of the bottlenose dolphin, no other cetacean species lives as long in captivity as in the wild.

For cetaceans, there appears to be a significant mismatch between the captive and natural environments in the amount and complexity of space available, socio-cultural opportunities, and cognitive stimulation between the captive and natural environments, which contributes to their difficulties in coping with life in concrete tanks (Mason, 2010; Hosey, Melfi & Ward, 2020). Additionally, the natural characteristics of cetaceans (*e.g.*, their need for space, cognitive and social complexity) predict the welfare challenges outlined above (Clubb & Mason, 2007; Pomerantz, Meiri & Terkel, 2013; Hosey, Melfi & Ward, 2020; Mellor et al., 2021). These outcomes are in keeping with Mason’s (2010) study of how different species respond to captivity. Mason (2010) concluded, in fact, that bottlenose dolphins and finless porpoises fare better in captivity than orcas, Fraser’s dolphins, or Dall’s porpoises. Much of the literature we have cited acknowledges areas where further study is needed. For instance, important questions remain about how the amount of space and complexity interact as factors in well-being, how to provide more supportive environments for nursing and rearing calves, and which enrichment methods are long-lasting and the most effective, and why. In addition, it is important to remember that the bulk of the cetacean welfare literature reviewed in the present manuscript, including the Cetacean Welfare Study, focuses on smaller cetaceans in accredited zoos/aquariums. There is much less welfare research on the larger odontocetes like orcas and beluga whales and virtually no research on unaccredited facilities. As such, one has to be careful in extrapolating current welfare findings to all cetaceans across different captive facilities, especially those that are unaccredited.

As noted in the introduction, there is a rapidly growing, largely unaccredited marine park industry in China. This situation has been summarized by Barefoot (2023, p. 284):

Whilst the practice of keeping marine mammals in aquaria is beginning to shift and decline in the west, it is at the same time growing in Asia, particularly China, which is experiencing an increase in captive facilities and trade in marine mammals. China’s number of aquatic theme parks grew from 39 in 2015 to over 80 in operation in 2019 with an additional 25 under construction. As of December 2020, China had over 1,000 cetaceans in captivity. Representatives from both the Chinese government and Chinese aquaria industry have expressed concerns with the rapid development of the industry and reliance on import of wild-caught animals, acknowledging that regulation has not maintained an appropriate pace.

More updated and much more detailed information can be found in a recent report (China Cetacean Alliance, 2024). Although this is not a peer-reviewed source, it provides valuable information about the current state of the Chinese marine park industry. The report notes that, as of July 2024, 101 ocean theme parks were operating in China, with 11 more under construction. Approximately 95 of these facilities offer cetacean shows involving tricks and trained behavior as well as close contact experiences for park visitors. The average size of the performance/exhibit tanks is 15.3 m length, 7.6 m wide, and 6.1 m depth. Over 1,300 cetaceans are currently in captivity in China (up from 184 in 2009; Zhang et al., 2012), including bottlenose dolphins (*n* = 738), beluga whales (*n* = 240), and orcas (*n* = 22). Since 2001, 1,166 cetaceans have been imported. A total of 83 births have been reported since 2002. It has been estimated that only ∼14 of these parks provide any kind of enrichment for the cetaceans. Many of the issues in the report mirror the welfare concerns discussed in the present review (*e.g.*, death of newborn/young calves, conspecific aggression, stereotypic behavior, logging, repetitive regurgitation, and several medical issues—gastrointestinal problems, fungal skin infections, worn down teeth). Thus, globally, significant welfare concerns remain for captive cetaceans. Even in the best, accredited facilities, there are significant (practical) limits to the extent to which land-based entertainment parks can provide larger, more complex, and variable environments that would allow cetaceans to engage in a greater range of natural behaviors. Pierce & Bekoff (2018) point out that discussions of animal welfare in zoos and marine parks often center on incremental improvements while overlooking the fundamental issue of captivity itself, namely the underlying incompatibility between captivity and life in the wild.

Welfare measures and assessments in accredited facilities comprise the bulk of the literature on captive animal welfare because one can collect scientific, quantifiable data. Such studies, however, do not address an important factor: quality of life. Presumably, this is because quality of life is a subjective evaluation made by the non-human animal and is not something one can scientifically measure in the absence of self-reporting. One can conduct choice and preference testing to help determine what is important to an animal (Dawkins, 2003), but the choices provided may not discern what the animal truly prefers, that is, what is actually optimal for the animal. For example, a researcher may give an animal a choice between food A and food Z, but what the animal prefers might actually be food B at time F, and food J at time Y. The former option provides the animal with a choice; the latter option is more reflective of true autonomy. In the Cetacean Welfare Study, “quality of life” is mentioned only one time: “There is a strong commitment among zoos and aquariums to continuously advance an understanding of welfare across facilities using scientific methods to positively impact the quality of life for the animal” (p. 2, Lauderdale et al., 2021b). As such, there is an assumption or belief that improved welfare measures will lead to a better quality of life (Pierce & Bekoff, 2018). However, the Cetacean Welfare Study did not evaluate the actual well-being of the dolphins; in fact, of the nine articles, there is only one mention of the term “well-being” (Miller et al., 2021c), and then only in a quote from Shepherdson (1998). The question thus remains: how can one definitively determine which welfare assessments actually provide quality of life? For example, researchers would probably agree that having autonomy (*i.e.,* choice and control over the environment) should improve well-being (Jaakkola, 2023) by providing captive animals with the opportunity to thrive (Vicino & Miller, 2015; Miller et al., 2020). But the relationship between autonomy and quality of life in a captive setting is not something one can easily quantify.

Although discussions of quality of life may verge into a philosophical realm, it remains a very real and tangible issue for those animals in captivity, and an important consideration when making decisions about animal welfare (Wolfensohn et al., 2015). An emphasis on quality of life leads to a focus on the positive mental experiences of individual animals (Green & Mellor, 2011). There are welfare measures now that attempt to consider quality of life. Here, we briefly highlight only two of them for illustrative purposes. Based on the Five Domains of animal welfare, the Animal Welfare Assessment Grid (AWAG) assesses physical health, veterinary and management issues, environmental comfort, and psychological well-being (Wolfensohn et al., 2015). Although originally developed for assessments in primates, AWAG is adaptable for any species (Wolfensohn et al., 2015). Specific to cetaceans, the Cetacean Welfare Assessment process (C-Well; Clegg, Borgner-Turner & Eskelinen, 2015) for captive bottlenose dolphins includes eleven criteria and a comprehensive collection of 36 species-specific measures. Like many such tools, these are works in progress. Nevertheless, they provide a foundation for more comprehensive assessment programs. A crucial consideration, however, is the extent to which such welfare measures are actually implemented. For the Cetacean Welfare Study, for example, apart from providing baseline reference measures through an iOS application (*e.g.*, blood variables, fecal hormone metabolites; Lauderdale et al., 2021b), it remains unclear if, to what extent, how, or where the welfare measures will be incorporated by captive facilities, and whether corrective action will be taken when deficiencies are identified. In recent years, there have been attempts to apply comprehensive assessment programs for dolphins (*e.g.*, the C-Well, Clegg, Borgner-Turner & Eskelinen, 2015; Dolphin-WET, Baumgartner et al., 2024). These efforts reflect growing progress toward embedding welfare assessments into the daily care of captive cetaceans (Jones et al., 2022).

From an ethical standpoint, the deeper question remains whether cetaceans can truly thrive in captivity, rather than merely survive. Thriving goes beyond physical health or lifespan—it encompasses overall *quality* of life and well-being. Partoon et al. (2025) suggest that a positive mental state is crucial to an animal thriving, and this involves promoting positive experiences for the animal through a species appropriate diet, naturally occurring social groupings, preventative healthcare, and a species appropriate habitat. Current evidence indicates that, although marine parks and aquariums can upgrade enclosures to offer some physical and behavioral benefit, they remain limited by available space and the artificial conditions required to keep cetaceans—especially larger species—in captivity. With few exceptions (*i.e.,* when individuals are captured from the wild or rescued and held in captivity for a short while), captive cetaceans cannot be released into the wild because they lack basic survival skills. Therefore, there are limited ethical alternatives. One of the most feasible options is a sanctuary, that is, ocean-based captive enclosures where cetaceans can continue to receive human care while experiencing more space, autonomy, and choice in a natural environment. There are currently three cetacean sanctuaries being created by the Whale Sanctuary Project, the National Aquarium, and SEALIFE TRUST (Marino et al., 2025). Authentic sanctuaries, as opposed to greenwashed or temporary sea pen facilities (Speiran, 2025), are still captive environments and therefore will share many management challenges with other captive facilities (*e.g.*, feeding, veterinary care, funding; Bruck, 2024). In particular, a cetacean born into captivity in a tank would likely experience a more challenging transition to an ocean sanctuary than one with experience in the wild or an ocean sea pen (Bruck, 2024). As in other aspects of welfare, it is necessary to be cognizant of individual differences among animals (Hill & Broom, 2009; Marchant-Forde, 2015). Nevertheless, a sanctuary has different tools to mitigate the challenges of captivity such as a larger, more natural environment that offers greater complexity and enhanced sensory-perceptual experiences. As opposed to zoos, marine parks, and other entertainment venues (as well as military and scientific settings) authentic sanctuaries are not driven by objectives that often compete with animal well-being and autonomy (*e.g.*, visitor experiences, scientific studies, breeding through AI). Accreditation standards for cetacean sanctuaries have been adopted by the Global Federation of Animal Sanctuaries (GFAS, 2025; Marino et al., 2025). Authentic sanctuaries for other wild animals, such as elephants and great apes, face many of the same challenges as cetacean sanctuaries but report improved physical and psychological health in their residents after they acclimate to their new environment (Buckley, 2009; Derby, 2009; Grow, 2020).

Although concerns about sanctuaries have been expressed by those who currently work with captive cetaceans (Bruck, 2024; Almunia & Canchal, 2025), it remains one of the only options available for those animals currently held in managed land-based tanks. Clearly, transitioning cetaceans to an authentic sanctuary requires careful planning (*e.g.*, priming the microbiota to buffer against novel pathogens; Dallas & Warne, 2023) and will require continued research to evaluate the welfare of transferred animals. Welfare assessments in sanctuaries will, in some ways, differ from those in zoos and aquariums as the animals will have access to and interact with more natural environments. The goal would be for animals to exhibit (1) greatly reduced or an absence of stereotypies, (2) greater engagement in natural behaviors, including those that were previously unavailable to them (*e.g.*, exploring/interacting with natural features of the environment, foraging), (3) increased time spent in play and other positive behaviors illustrating greater behavioral diversity, (4) time budgets closer to what exists in nature, (5) decreased time spent in human-animal interactions, (6) increased autonomy and the ability to make meaningful choices, and (7) a decrease in physiological indicators of stress. These goals are consistent with the five domains model of animal welfare (Hampton et al., 2023; Partoon et al., 2025). Although none of these, in isolation, indicates that an animal is thriving, the broad confluence of all these factors would indicate more natural behavior and would help attain the goals set by accrediting agencies mentioned previously, namely: “maximize psychological health” (WAZA, 2025), stimulate “natural behavior” (Association of Zoos & Aquariums (AZA), 2025), allow the animal “variety and choices” in their environment (Alliance for Marine Mammal Parks Aquariums (AMMPA), 2025), and to provide the animal with “behavioural choices” and “control over its environment” (EAAM, 2019).

In conclusion, the evidence clearly shows that ongoing health and welfare challenges remain for captive cetaceans, indicating that they are generally unsuited for captivity from both practical and ethical standpoints. Marine parks, particularly those that are accredited and have environmental enrichment programs, have improved in their ability to provide better welfare for captive cetaceans, especially the smaller ones like bottlenose dolphins. Nevertheless, they still cannot fully meet the complex needs of these animals. As interest and experience in captive animal well-being grows, it is essential to acknowledge when certain environments fail to provide what a species needs to thrive. Moving forward, science-based policies should be considered to determine which species should no longer be housed or bred in zoos, aquariums, and entertainment parks.

## Additional Information and Declarations

### Data Availability

The following information was supplied regarding data availability: This is a literature review hence it did not utilize raw data.
